# Supplementary material for: Effect of Contamination by Phosphate Mining Effluent on Biocrust Microbial Community Structure and Cyanobacterial Diversity in a Hot Dry Desert
Source: Microorganisms. 2025 Nov 12;13(11):2580. doi: 10.3390/microorganisms13112580 (PMC12654040; doi:10.3390/microorganisms13112580)
Supplement: Supplementary file 1 [file microorganisms-13-02580-s001.zip › microorganisms-3970421-supplementary.pdf]

Supplementary Materials

Effect of contamination by phosphate mining effluent on biocrust microbial community structure and cyanobacterial diversity in a hot dry desert

Ali Nejidat[1,*], Damiana Diaz-Reck[1], Eli Zaady[2]

[1]Department of Environmental Hydrology and Microbiology, Zuckerberg Institute for Water Research, The Jacob Blaustein Institutes for Desert Research, Ben-Gurion University of the Negev, Sede Boqer Campus 84990, Israel.

[2]Katif Research & Development Center, The Ministry of Innovation, Science and Technology, Netivot 8771002, Israel.

Table S1: Soil mineral content. Values in the table mean mg g^{-1} dry soil ± SD of 4 replicates. B, Cd, Co, Li, Ni, Pb, Se and Zn were below the limit of detection. Soil samples (top 5 cm) collected in November 2018.

	**Ashalim 1**					**Ashalim 2**					**Control- Gmalim**		
	**Stream bed**	**Dark soil**	**Bright soil**	**OM foam**	**Stream banks**	**Stream bed**	**Dark soil**	**Bright soil**	**OM foam**	**Stream banks**	**Stream bed**	**Shrubs**	**Stream banks**
**Element**	**A1G**	**A1D**	**A1L**	**A1O**	**A1N**	**A2G**	**A2D**	**A2L**	**A2O**	**A2N**	**GG**	**GS**	**GN**
**Al**	**2.1±0.1**	**5.3±4.9**	**6.3±0.0**	**7.1±4.2**	**17.5±1.5**	**5.5±2.5**	**10.6±0.0**	**8.5±0.1**	**11.6±2.0**	**9.8±2.7**	**3.2±0.6**	**13.2±0.7**	**16.2±1.8**
Ba	0	0.1±0.0	0	0.1-0.0	0.1±0.0	0	0.1±0.0	0.1±0.0	0.2±0.1	0.3±0.0	0	0.1±0.0	0.1±0.0
**Ca**	**11.4±0.0**	**61.2±29.1**	**41.5±2.4**	**225.8±53**	**113.0±6.5**	**41.6±18.1**	**64.0±0.0**	**59.9±4.6**	**159.5±13**	**162.4±41**	**9.1±1.6**	**72.1±6.1**	**90.5±2.7**
Cr	0	0.04±0.02	0.01±0.0	0.02±0.01	0.02±0.01	0.01±0.01	0.03±0.0	0.02±0.0	0.04±0.02	0.02±0.0	0.01±0.0	0.02±0.0	0.02±0.0
Cu	0	0.01±0.01	0	0.01±0.0	0.01±0.0	0	0.01±0.0	0.01±0.0	0.01±0.01	0.01±0.0	0	0.01±0.0	0.01±0.0
**Fe**	**1.2±0.2**	**5.3±2.4**	**4.1±0.0**	**4.3±3.4**	**10.5±2.7**	**3.2±1.7**	**6.5±0.0**	**5.7±0.3**	**4.4±3.2**	**4.8±1.7**	**1.5±0.3**	**7.3±0.3**	**10.1±1.4**
K	0.4±0.0	2.5±1.1	1.4±0.0	1.7±0.8	3.1±0.3	1.2±0.6	2.4±0.0	2.0±0.0	2.4±0.4	2.2±0.5	0.5±0.1	2.1±0.0	3.3±0.2
**Mg**	**0.3±0.0**	**1.9±0.7**	**2.6±0.1**	**4.5±1.5**	**8.7±0.4**	**1.5±1.0**	**4.2±0.0**	**3.8±0.2**	**6.7±0.0**	**6.5±1.2**	**0.9±0.2**	**5.0±0.2**	**8.2±0.2**
Mn	0	0.1±0.0	0.1±0.0	0.1±0.0	0.2±0.0	0	0.1±0.0	0.1±0.0	0.2±0.0	0.1±0.0	0	0.1±0.0	0.2±0.0
Na	0.1±0.0	0.7±0.0	0.6±0.1	0.3±0.2	1.6±0.0	0.6±0.3	0.9±0.0	0.7±0.0	0.7±0.1	0.6±0.2	0.1±0.0	0.4±0.1	0.8±0.2
P	0.9±0.1	4.9±0.0	3.1±0.2	1.2±0.0	2.5±1.3	1.9±0.0	5.4±0.0	3.5±1.0	3.9±0.0	4.0±0.0	0.6±0.1	4.2±0.6	1.6±0.3
S	1.7±0.4	2.6±0.0	1.1±0.1	3.8±0.2	2.0±0.2	4.9±0.0	2.9±0.0	2.0±0.0	3.9±1.2	2.7±0.6	0.2±0.0	1.2±0.1	1.5±0.1
Si	0.1±0.0	1.1±1.0	0.2±0.0	1.0±0.4	0.7±0.4	0.2±0.1	0.6±0.0	0.4±0.2	0.5±0.0	0.5±0.4	0.3±0.0	0.8±0.0	0.8±0.3
Sr	0.1±0.0	0.2±0.0	0.1±0.0	0.3±0.0	0.2±0.0	0.2±0.1	0.2±0.0	0.2±0.0	0.4±0.1	0.3±0.0	0	0.2±0.0	0.2±0.0
**Total**	**18.3**	**85.95**	**61.11**	**250.23**	**160.13**	**60.81**	**97.94**	**86.93**	**194.45**	**194. 23**	**16.41**	**106.73**	**133.53**

Table S2 (November 2018 samples): Relative abundance (%) of the assigned **bacterial phyla**, based on valid sequences of all OTUs belonging to the same phylum (after subtracting non-assembled reads, and discarding of short and chimera sequences). Relative abundance was calculated by summing the clean sequences of all OTUs belonging to each phylum divided by the total number of all clean DNA sequences generated for the sample and multiplied by 100. Values are averages of 2 separate amplicons each containing a mix of PCR products extracted from two composite (3 subsamples) soil samples. Sites: G, Gmalim; A1, Ashalim site 1; A2, Ashalim site 2. Ashalim Strips: G, stream bed; D, dark soil surface; L, bright soil surface; O, organic matter surface; N, stream bank surface. Gmalim strips: G, stream bed; S, shrub area; N, stream bank.

Phylum	Control- Gmalim (Layers)			Ashalim- Site 1					Ashalim- Site 2				
	Stream bed	Shrubs	Stream banks	Stream bed	Dark soil	Bright soil	OM foam	Stream banks	Stream bed	Dark soil	Bright soil	OM foam	Stream banks
	**GG**	**GS**	**GN**	**A1G**	**A1D**	**A1L**	**A1O**	**A1N**	**A2G**	**A2D**	**A2L**	**A2O**	**A2N**
Actinobacteria	15.0	18.5	25.5	14.5	29	24.5	27.5	27.0	20.0	40.0	13.5	47.0	32.0
Proteobacteria	33.0	32.5	27.0	46.0	35.5	27.5	28.5	23.5	46.0	26.0	31.0	15.5	32.0
Bacteroidetes	16.5	18.0	11.5	23.5	14.5	16.5	16.0	14.0	22.5	6.0	3.5	3.5	9.5
Cyanobacteria	20.0	6.0	7.0	2.0	1.5	0	1.0	1.0	1.0	0	0	0	0
Firmicutes	1.5	3.5	1.0	1.5	15	14.5	8.5	1.0	2.5	8.5	49.0	10.5	1.0
Chloroflexi	4.0	6.0	13.5	2.5	2.0	2.0	10.5	19.0	2.5	7.0	0.5	12	12.5
Gemmatimonadetes	2.0	3.0	3.0	2.5	0.5	1.0	1.0	3.5	1.0	5.0	0	3.0	3.0
Acidobacteria	2.0	3.5	3.0	0.5	0.5	0.5	0.5	1.0	0	1.0	1.0	0	1.0
Planctomycetes	1.0	2.0	1.5	0.5	0	0	0	0.5	0	0.5	0	1.0	1.0
Patescibacteria	1.5	2.0	1.0	3.0	1.5	4.5	4.5	2.5	1.5	3.5	0	1.0	2.0
Deinococcus-Thermus	1.0	1.0	1.0	2.0	0.5	0	1.5	6.5	3.5	1.0	1.5	1.5	3.0
Verrucomicrobia	1.0	2.0	2.0	0.5	0	0	0.5	0.5	0	0	0	0	1.0
**Total (%)**	98.5	98	97	99	100.5	91	100	100	100.5	98.5	100	95	98
**Valid sequences (2 repeats)**	**71419-74598**	**95035-89486**	**80680-84986**	**86769-73441**	**78611-77296**	**84056-92897**	**95718-97413**	**108828-92713**	**55407-78111**	**61483-51057**	**40566-12245**	**63278-67717**	**80196-56639**

Table S3 (July 2022 samples): Relative abundance (%) of assigned **bacterial phyla** based on valid sequences of all OTUs belonging to the same phylum (after subtracting non-assembled reads and discarding of short and chimera sequences). Relative abundance was calculated by summing the clean sequences of all OTUs belonging to each phylum divided by the total number of all clean DNA sequences generated for the sample and multiplied by 100. An average of 2 separate amplicons each containing contains a mix of PCR products extracted from two composite (3-subsamples) soil samples Sites: G, Gmalim; A1, Ashalim site 1; A2, Ashalim site 2. Ashalim Strips: G, stream bed; D, dark soil surface; L, bright soil surface; O, organic matter surface; N, stream bank surface. Gmalim strips: G, stream bed; S, shrub area; N, stream bank.

Phylum	Control- Gmalim (Strips)			Ashalim- Site 1					Ashalim- Site 2				
	Stream bed	Shrubs	Stream banks	Stream bed	Dark soil	Bright soil	OM foam	Stream banks	Stream bed	Dark soil	Bright soil	OM foam	Stream banks
	GG	GS	GN	A1G	A1D	A1L	A1O	A1N	A2G	A2D	A2L	A2O	A2N
Actinobacteria	17.8	10.0	17.8	14.3	43.5	19.0	13.1	17.9	13.6	14.7	21.1	22.0	13.3
Proteobacteria	21.5	12.4	36.8	38.8	16.9	19.9	27.8	24.0	39.8	25.8	35.1	28.4	26.0
Bacteroidetes	21.1	37.6	9.9	23.2	19.5	18.4	22.9	18.9	26.1	31.7	21.7	14.6	18.7
Chloroflexi	13.6	11.7	13.6	3.9	4.3	7.6	12.2	12.5	3.4	9.3	4.5	21.8	16.2
Cyanobacteria	13.0	10.1	2.5	1.6	0.2	0.1	0.2	1.1	0.4	0.17	0.2	0.1	0.3
Firmicutes	0.8	5.9	3.2	8.6	7.3	16.5	12.1	12.0	10.1	6.5	2.7	1.2	7.7
Acidobacteria	4.5	2.5	2.8	0.6	0.1	0.2	0.6	0.3	0.4	0.17	0.2	2.8	2.1
Deinococcota	3.5	4.8	9.3	1.4	1.3	1.6	3.9	8.4	0.8	2.7	1.9	4.4	8.3
Gemmatimonadota	1.0	0.4	0.9	1.5	1.0	0.7	0.4	0.6	0.4	0.59	0.6	1.1	0.9
Patescibacteria	0.7	3.2	0.4	3.9	5.3	14.3	4.6	2.7	2.7	7.4	10.5	2.4	4.9
Verrucomicrobiota	1.5	0.7	1.9	0.4	0.0	0.5	1.2	0.5	0.7	0.24	0.3	0.6	0.9
Bdellovibrionota	0.3	0.2	0.2	1.3	0.6	0.6	0.5	0.9	0.7	0.3	0.8	0.2	0.3
**Total**	**99.3**	**99.5**	**99.3**	**99.5**	**99.5**	**99.5**	**99.5**	**99.7**	**99.1**	**99.57**	**99.6**	**99.5**	**99.5**
**Valid sequences (2 repeats)**	**13134-8977**	**23684-17263**	**27541-12050**	**50700-37731**	**56213-76395**	**29969-30029**	**27412-20798**	**32331-33043**	**46719-45630**	**25774--**	**29608-44600**	**29436-31919**	**15546-30196**

Table S4 (November 2018 samples): Relative abundance (%) of the identified **dominant genera** within each dominant phylum based on valid sequences (after subtracting non-assembled reads, discarding of short and chimera sequences) of all assigned genera. Results are an average of 2 repeats and each repeat contained a combined DNA extracted from two composite biocrust samples. Given are the genera of phyla having at least one genus with 1% abundance in any of the samples. OM -organic matter.

Phylum	Genus	Control- Gmalim (Strips)					Ashalim- Site 1			Ashalim- Site 2				
		Stream bed	Shrubs	Stream banks	Stream bed	Dark soil	Bright soil	OM foam	Stream banks	Stream bed	Dark soil	Bright soil	OM foam	Stream banks
Acidobacteria	*Bryobacter*	0.93	1.71	0.93	0.13	0.12	0.02	0.11	0.20	0.04	0.15	0.19	0.02	0.46
	Blastocatella	0.18	0.83	0.54	0.08	0.09	0.00	0.13	0.36	0.02	0.01	0.00	0.01	0.22
Actinobacteria	*Blastococcus*	0.80	1.22	1.26	1.79	4.92	9.34	2.54	1.26	3.18	2.26	3.39	6.94	2.19
	Geodermatophilus	0.69	0.54	1.72	0.28	1.52	1.97	0.39	0.77	0.42	1.78	0.11	1.22	1.07
	Modestobacter	0.20	0.19	0.21	0.72	2.30	0.37	0.55	0.36	1.03	0.59	0.76	0.25	0.23
	Arthrobacter	3.61	2.73	1.36	3.99	1.62	4.48	6.23	3.00	7.69	4.48	3.50	5.11	1.66
	Kocuria	0.20	0.89	0.19	0.13	0.25	1.60	0.32	0.29	0.36	3.70	0.62	0.76	0.85
	Pseudarthrobacter	1.74	3.14	1.52	1.54	9.16	4.53	2.96	1.34	1.54	2.11	1.59	2.66	2.35
	Marmoricola	0.52	0.49	0.28	0.14	2.05	1.37	0.43	0.22	0.22	2.58	0.25	0.67	0.50
	Rubrobacter	1.37	2.45	9.28	1.14	0.35	0.39	5.63	9.48	1.19	5.17	0.55	9.25	8.55
	Solirubrobacter	0.26	0.28	0.88	0.25	0.07	0.06	0.69	0.69	0.22	1.26	0.06	0.92	1.22
Bacteroidetes	*Flavisolibacter*	1.38	1.77	1.51	1.60	0.81	0.56	0.53	0.34	0.48	0.33	0.01	0.03	0.67
	Segetibacter	1.54	2.76	2.52	0.38	0.16	0.02	0.85	0.50	0.08	0.04	0.11	0.03	0.70
	Rhodocytophaga	2.51	2.08	1.96	0.73	0.64	0.01	1.07	1.45	0.87	0.14	0.14	0.20	0.98
	Adhaeribacter	4.77	2.77	2.18	4.08	1.72	0.40	6.20	1.64	4.76	0.76	1.11	0.99	1.99
	Hymenobacter	1.18	0.69	0.47	0.60	0.22	0.03	0.56	0.15	2.24	0.14	0.18	0.12	0.28
	Nibribacter	0.13	0.09	0.03	0.18	0.02	0.05	0.31	0.16	0.32	0.07	0.03	0.09	0.02
	Pontibacter	0.60	3.64	1.10	6.00	6.35	13.24	3.70	8.40	9.86	1.37	0.93	1.01	3.41
	Pedobacter	0.14	0.05	0.02	1.05	0.64	0.57	0.11	0.00	0.26	0.43	0.31	0.05	0.01
Chloroflexi	*Kallotenue*	0.64	0.43	0.94	0.13	0.11	0.00	0.34	1.39	0.13	0.07	0.17	0.46	0.78
Deinococcus-Thermus	*Deinococcus*	0.42	0.12	0.23	1.10	0.48	0.04	0.52	0.24	1.91	0.15	0.29	0.45	0.23
	Truepera	0.58	1.01	0.85	0.79	0.24	0.03	1.01	6.24	1.58	0.59	0.16	1.09	2.81
Firmicutes	*Bacillus*	0.19	0.15	0.24	0.23	2.68	5.28	0.23	0.14	0.35	6.48	15.31	0.93	0.11
	Paenibacillus	0.09	0.06	0.04	0.13	0.68	0.12	0.05	0.01	0.07	1.03	3.96	0.00	0.02
	Planococcus	0.28	0.33	0.40	0.28	0.24	1.08	0.92	0.17	0.17	0.00	0.02	0.52	0.06
	Planomicrobium	1.00	2.40	0.38	0.96	1.02	7.51	6.92	0.63	1.26	0.11	0.90	9.95	0.74
	Pullulanibacillus	0.00	0.00	0.00	0.04	5.09	0.03	0.00	0.00	0.26	0.12	31.15	0.00	0.00
Proteobacteria	*Skermanella*	1.77	1.65	1.75	2.46	0.94	1.24	1.48	1.16	2.87	0.30	0.55	1.05	1.22

	Microvirga	5.33	6.08	6.39	1.82	1.44	7.41	3.67	3.21	1.47	1.61	0.79	2.68	5.98
	Rubellimicrobium	7.31	3.66	2.63	5.00	2.50	0.13	3.38	2.05	4.74	1.12	1.08	0.95	4.12
	Ellin6055	2.13	3.31	3.47	3.20	1.09	1.66	3.96	4.44	1.54	1.33	0.60	2.32	5.39
	Novosphingobium	0.24	1.73	0.02	0.38	0.28	0.17	0.19	0.03	0.26	0.16	0.80	0.06	0.10
	Sphingomonas	2.19	2.05	1.62	4.60	2.18	1.47	2.28	1.89	2.39	2.16	0.38	1.46	2.33
	Massilia	0.52	0.18	0.20	10.59	11.1	4.97	3.69	0.31	16.05	3.95	10.83	4.11	0.24
	Noviherbaspirillum	1.49	0.83	0.99	4.59	5.86	4.77	2.96	1.59	8.79	3.25	0.82	1.48	1.06
	Nitrosomonas	0.40	0.11	0.19	0.02	0.03	0.00	0.05	0.05	0.01	0.00	0.02	0.00	0.12
	Azospirillum	0.000377	0.00057	0.00043	0.0004	0.0014	0.0013	0.0005	0.0001	0.0006	0.0003	0.0138	0.0004	0.0003
Verrucomicrobia	*Candidatus_ Udaeobacter*	0.18	1.26	2.02	0.11	0.02	0.00	0.43	0.62	0.01	0.00	0.03	0.08	0.78

Table S5 (July 2022 samples): Relative abundance (%) **of identified genera** within each dominant phylum based on valid sequences (after subtracting non-assembled reads, discarding of short and chimera sequences) of all assigned genera. Results are an average of 2 repeats and each repeat contained combined DNA extracted from two composite biocrust samples. Given are the genera of phyla having at least one genus with 1% abundance in any of the samples. OM - organic matter.

		Control- Gmalim (Layers)			Ashalim- Site 1					Ashalim- Site 2				
**Phylum**	***Genus***	**Stream bed**	**Shrubs**	**Stream banks**	**Stream bed**	**Dark soil**	**Bright soil**	**OM foam**	**Stream banks**	**Stream bed**	**Dark soil**	**Bright soil**	**OM foam**	**Stream banks**
Acidobacteria	*Bryobacter*	3.43	1.21	0.83	0.18	0.11	0.15	0.16	0.02	0.10	0.07	0.04	0.65	0.14
Actinobacteria	*Blastococcus*	0.84	1.54	0.25	2.52	5.23	3.55	0.70	0.88	1.53	1.10	2.50	1.38	0.43
	uncultured	0.33	0.28	0.60	1.43	1.18	0.57	0.46	2.57	0.76	0.45	1.14	0.70	0.58
	Arthrobacter	1.45	0.67	1.83	1.87	1.01	2.35	2.93	5.73	3.08	3.20	3.11	2.90	2.87
	Kocuria	0.26	0.22	0.43	0.66	0.41	0.00	0.40	1.00	0.87	0.19	0.54	0.32	1.42
	Pseudarthrobacter	6.35	2.65	3.13	2.15	4.38	2.62	3.22	2.80	2.87	2.45	5.36	1.51	1.08
	Rubrobacter	2.54	3.57	9.11	1.01	0.58	3.16	3.20	1.78	0.48	1.79	1.80	8.08	3.96
Bacteroidetes	*Flaviaesturariibacter*	0.61	3.45	0.29	1.01	0.72	0.21	0.56	0.76	0.88	0.34	0.44	0.04	0.50
	Segetibacter	0.35	11.52	1.23	0.13	0.36	0.34	0.65	0.21	0.20	0.32	0.72	0.30	0.25
	Rhodocytophaga	3.94	2.54	1.66	1.57	1.25	1.97	1.79	1.47	1.03	0.95	1.19	2.63	1.57
	Adhaeribacter	2.14	5.27	3.48	4.95	2.72	3.54	7.47	2.40	6.91	6.48	7.34	4.50	1.73
	Nibribacter	0.00	0.09	0.01	0.12	0.06	0.11	0.11	0.13	0.39	2.01	1.67	0.00	0.00
	Pontibacter	14.99	11.30	1.34	11.77	6.87	9.94	12.1	12.96	12.6	19.28	7.54	7.17	9.37
	Rufibacter	0.37	0.34	0.22	0.36	0.19	0.06	0.38	0.39	0.78	2.97	0.50	0.11	0.05
	_Arcticibacter	0.06	0.00	0.11	0.60	0.97	0.10	0.10	0.00	1.23	0.12	0.28	0.03	0.03
Chloroflexi	*AKIW781*	6.52	6.38	8.87	1.75	2.73	1.48	5.98	4.96	1.06	3.28	1.52	11.3	4.00
	AKYG1722	1.67	1.28	0.68	0.28	0.44	1.55	0.97	0.94	0.33	1.27	0.65	1.03	2.13
	JG30-KF-CM45	3.84	4.55	2.26	1.81	2.62	2.52	4.12	2.25	1.71	3.74	1.79	4.06	4.47
Deinococcota	*Deinococcus*	0.38	0.02	0.41	0.23	0.23	0.98	0.44	1.13	0.14	0.62	0.64	0.41	0.19
	Truepera	3.38	5.22	9.48	1.29	0.65	0.80	4.01	7.96	0.72	2.34	1.35	4.19	8.93
Firmicutes	*Bacillus*	0.20	0.00	0.00	1.54	5.87	10.76	0.03	1.18	0.39	0.00	0.45	0.06	0.39

	Planococcus	0.00	3.55	0.67	0.47	0.27	0.00	1.70	4.40	1.26	0.17	0.07	0.00	2.08
	Planomicrobium	0.45	0.34	0.25	0.55	0.27	0.00	1.21	0.16	0.90	0.28	0.04	0.00	0.00
Patescibacteria	*LWQ8*	0.25	0.54	0.12	1.10	1.71	4.35	1.74	0.76	0.77	1.18	2.34	0.68	2.09
	TM7a	0.02	0.70	0.04	1.26	1.31	0.49	0.50	0.34	0.88	2.25	1.34	0.19	0.08
	Saccharimonadales	0.25	1.63	0.18	1.49	1.58	7.97	2.30	1.60	1.12	4.03	5.78	1.10	2.67
Proteobacteria	*Skermanella*	1.45	0.00	1.24	3.87	1.96	1.57	1.73	0.59	2.22	4.02	2.80	0.89	1.08
	Microvirga	7.68	0.00	12.37	7.20	5.84	3.72	7.20	3.72	7.23	2.29	3.28	6.12	5.47
	Devosia	0.74	0.00	0.14	3.67	2.20	0.92	1.77	0.91	4.94	1.01	2.16	0.82	2.21
	uncultured	0.85	0.60	0.62	0.57	0.32	0.64	1.30	0.39	0.62	0.21	0.56	2.63	2.51
	Paracoccus	0.66	4.04	0.02	0.61	0.32	0.11	0.52	0.55	0.79	0.37	1.30	0.11	0.60
	Rubellimicrobium	6.87	5.84	9.83	8.38	4.66	2.42	5.95	5.47	6.79	5.52	7.22	5.68	2.93
	Sphingomonas	0.50	0.00	1.73	3.78	2.67	0.73	1.62	2.25	3.57	1.15	1.90	2.04	2.39
	Nitrosospira	0.00	0.00	0.02	0.05	1.43	1.13	0.04	0.05	0.09	0.05	0.25	0.08	0.15
	Azospirillum	0	0	0	0.0013	0.09	0	0.005	0.0008	0.002	0.0013	0.0007	0	0.0003
